# Conditioned Medium Derived From Human Dental Follicle Mesenchymal Stem Cells Alleviates Macrophage Proinflammatory Responses Through MAPK-ERK-EGR1 Axis

**DOI:** 10.1155/sci/5514771

**Published:** 2024-11-29

**Authors:** Chuhan Zhang, Peiyi Lv, Qiuying Liang, Jian Zhou, Buling Wu, Wenan Xu

**Affiliations:** ^1^Shenzhen Clinical College of Stomatology, School of Stomatology, Southern Medical University, Guangzhou, China; ^2^Shenzhen Stomatology Hospital (Pingshan), Southern Medical University, Shenzhen, China; ^3^Salivary Gland Disease Center and Beijing Key Laboratory of Tooth Regeneration and Function Reconstruction, Beijing Laboratory of Oral Health and Beijing Stomatological Hospital, Capital Medical University, Beijing, China

## Abstract

The regulation of macrophage polarization by mesenchymal stem cells (MSCs) is a prominent area of research but faces challenges due to limited MSC sources and incomplete understanding of underlying mechanisms. We sought to identify an accessible MSC source and investigate how MSCs regulate macrophage polarization using high-throughput sequencing. We isolated dental follicle MSCs from discarded human third molar dental follicles and cocultured them with THP-1-derived macrophages in the conditioned medium. Transcriptome sequencing identified differentially expressed genes (DEGs) in macrophages, integrating with multiomics database analysis to uncover polarization mechanisms. Our findings demonstrated successful MSC extraction from dental follicles, with the conditioned medium suppressing proinflammatory macrophage functions and influencing macrophage subtyping. MSCs, through paracrine signaling, activated the mitogen-activated protein kinase (MAPK) pathway, leading to extracellular regulated protein kinases (ERK)1/2 phosphorylation and upregulation of early growth response 1 (EGR1) protein. Elevated EGR1 levels inhibited inflammatory gene expression, inhibiting the pro-inflammatory immunoregulatory function of macrophages in inflammatory states. This study provides an efficient method for in vitro macrophage polarization identification. It offers insights into MSC-regulated polarization mechanisms, with potential clinical implications for anti-inflammatory therapy and immune regulation.

## 1. Introduction

Macrophages, or M*φ*, are pivotal in immune responses and can differentiate into M1 and M2 subtypes [1, 2]. M1 macrophages, characterized by CD86 and CD80 as their surface markers, primarily contribute to proinflammatory processes, releasing harmful mediators and exacerbating inflammation. In contrast, M2 macrophages, characterized by CD163 and CD206, exhibit anti-inflammatory properties and aid tissue repair [1, 3, 4]. The balance between M1 and M2 macrophage polarization significantly impacts the outcome of organs or tissues during inflammation or injury [3, 5–7]. Consequently, modulating this delicate balance has become a research focus for potentially altering the course of inflammatory diseases through physical environmental influences, chemical interventions, and various biological factors [7].

Since the initial isolation from human bone marrow in 1992, mesenchymal stem cells (MSCs), a diverse group of multipotent stem cells, have garnered considerable attention due to their extensive therapeutic applications [8–10]. MSCs have been shown to exert regulatory effects on macrophages through various mechanisms, such as direct cell–cell interaction and the secretion of soluble factors [11–13]. The release of numerous bioactive molecules, including transforming growth factor *β* (TGF-*β*), chemokine CCL12, and chemokine ligand CXCL12, actively modulates the immune function of macrophages through the soluble factor pathway [9, 14]. Overall, the secretome of the MSC, consisting of proteins secreted into the extracellular space, plays a vital role in cell-to-cell communication and possesses immunomodulatory properties [15, 16]. Dental follicle-derived MSCs (DFMSCs), obtained from the soft tissue surrounding the unerupted tooth crown, have emerged as a promising alternative due to their wide availability and low ethical controversy [17–21]. DFMSC exhibits superior proliferation, multidirectional differentiation, and immunomodulatory potential compared to other dental-derived stem cells. Recent studies have demonstrated their superior immunomodulatory effects, offering potential opportunities for DFMSC-based cell therapy in immune-related diseases [21–25]. Despite progress in utilizing DFMSC for immunomodulation, there remains a significant knowledge gap regarding the composition and immunoregulatory functions of both MSC's and DFMSC's secretomes [13, 26]. A comprehensive understanding of the secretome's immunomodulatory effects and underlying mechanisms is crucial for optimizing DFMSC-based therapies and developing novel strategies to regulate macrophage polarization. Therefore, this study aims to investigate the immunomodulatory effects of the DFMSC secretome and elucidate its mechanisms of action.

In this study, we chose to coculture THP-1-derived macrophages with DFMSC conditioned medium (DFMSC-CM) under inflammatory conditions. Morphological changes in polarized macrophages were confirmed by dual-color cell immunofluorescence staining for the first time. Furthermore, we were able to accurately determine the molecular mechanisms DFMSC-CM primarily acted through the mitogen-activated protein kinase (MAPK)-extracellular regulated protein kinases (ERK)1/2-early growth response 1 (EGR1) pathway by utilizing transcriptomic sequencing. By addressing this scientific question, we establish a solid theoretical foundation for enhancing the efficacy of DFMSC in inflammation-immune regulation and provide novel insights into the regulation of macrophage polarization by MSCs.

## 2. Materials and Methods

### 2.1. Reagents

Cell culture-related reagents, including Dulbeccoʼs modified eagle medium (DMEM)/F12, RPMI-1640, phosphate buffer solution (PBS), tris buffered saline (TBS), penicillin/streptomycin (P/S), and fetal bovine serum (FBS), are from GIBCO (USA). Trypsin/ethylene diamine tetraacetic acid (EDTA) was obtained from Vivacell (China). Differentiation induction kits, OriCell Human Stem Cells Lipid-induced Differentiation Kit and OriCell Human Stem Cell Chondrogenic Induced Differentiation Kit were from Cyagen (China). Dexamethasone, L-ascorbic acid, and *β*-glycerophosphate for osteogenic differentiation and L-glutamine, phorbol 12-myristate 13-acetate (PMA), lipopolysaccharide (LPS), and interferon (IFN)-*γ* were from Sigma (USA). Flow cytometry antibodies were from BD biosciences (USA). Cell Counting Kit-8 (CCK8) was provided by DOJINDO (Japan). Quantitative real-time polymerase chain reaction (PCR) (qRT-PCR) reagents were from EzBioscience (USA). Primary antibodies for immunostaining (CD68, CD80) were from Abcam (USA). An anti-EGR1 antibody from Abcam was used for the western blot and immunostaining. CD206 Polyclonal antibody was from Proteintech (China). Primary antibodies for western blot (glyceraldehyde-3-phosphate dehydrogenase [GAPDH], Lamin A/C) were also from Proteintech. MAPK-related protein antibodies were from CST's MAPK Family Antibody Sampler Kit. Fluorescent secondary antibodies were provided by BOSTER (China). Nuclear Extract Kit used for protein extraction was from Active Motif (USA). An antifluorescence quenching sealing solution and a BeyoECL Plus Ultrasensitive Kit were provided by Beyotime (China).

### 2.2. Cell Culture and Identification

#### 2.2.1. Culture of DFMSC

According to the guidelines of the Ethics Committee at Southern Medical University Shenzhen Stomatology Hospital (Pingshan), tissue samples were collected from clinical volunteers. Tissue was used to obtain DFMSC in DMEM/F12 medium supplemented with 1% P/S and 10% FBS at 37°C with 5% CO_2_. The medium was changed every 2–3 days. Cell migration was observed, and upon monolayer formation, they were passaged using trypsin/EDTA. DFMSC in passages 3–5 were used for subsequent experiments.

#### 2.2.2. Adipogenic Differentiation

The premixed lipid-induced medium from the lipid-induced differentiation kit was used for 1 day of adipogenic induction and 3 days of adipogenic induction maintenance medium cultivation. This was repeated in a cycle for 28 days of induction cultivation. Oil Red O staining was used to observe adipocytes and lipid droplets in the cells.

#### 2.2.3. Osteogenic Differentiation

Cells were seeded in 12-well plates at a density of 2 × 10^5^ cells per well. The next day, cells were washed and induced with osteogenic medium containing 1% P/S, 1 mM dexamethasone, 10 nM L-ascorbic acid, 1M *β*-glycerophosphate, and 10% FBS. The medium was refreshed every 3 days for 21 days. Mineralized crystal was stained by Alizarin Red S staining.

#### 2.2.4. Chondrogenic Differentiation

5 × 10^5^ cells were washed and centrifuged in a 15-mL tube. The pellet was resuspended in a 0.5 mL chondrogenic induction medium. After centrifuging at 1000 rpm for 5 min, cells were cultured at 37°C, 5% CO_2_. Spherical cell pellets were formed after 24 h. Medium with TGF-*β*3 was changed every 3 days for 21–28 days. Cell pellets were embedded, sectioned, and stained with Alcian blue.

#### 2.2.5. Culture and Induction of THP-1 Cells

The THP-1 cell line was obtained from ATCC (USA). The EGR1 knockout cell line constructed using Clustered Regularly Interspaced Short Palindromic Repeats (CRISPR)/Cas9 was purchased from EDITGENE (China). Cells were cultured in RPMI-1640 medium supplemented with 1% P/S, 1% L-glutamine, and 10% heat-inactivated FBS. After seeding THP-1 cells at a 1 × 10^6^ cells/mL density in six-well plates, macrophages were derived after a 48-h light-protected induction with 50 ng/mL PMA. M1 macrophages were obtained by further stimulating the derived macrophages with 100 ng/mL LPS and 20 ng/mL IFN-*γ* for 24 h.

#### 2.2.6. Flow Cytometry

P3-P5 DFMSC were washed twice and resuspended in 100 μL at a concentration of 1 × 10^6^ cells. The cells were stained with CD90-PE, CD105-PE, CD73-APC, CD45-V500, and CD34-PE antibodies for 30 min at 4°C in the dark. Adherent cells were dissociated as a single-cell suspension to assess the successful induction of THP-1 cells into macrophages. The cells were then incubated in the dark with CD11b-PE-Cy7 antibody. All cells were analyzed by flow cytometry.

### 2.3. Collection of DFMSC-CM

DFMSC from passages 3–5 were cultured in 75 mm^2^ flasks. DFMSC-CM was collected at cell densities of 50%–100%. RPMI-1640 medium without serum was added at volumes of 10, 15, and 20 mL for 24 or 48 h. The supernatant was centrifuged, filtered, and concentrated to 20% of the original volume using 3 kDa ultrafiltration tubes (Millipore, USA) at 4°C. The protein concentration of the CM was determined, and DFMSC cell viability was assessed to establish a standardized extraction protocol.

### 2.4. Protein Concentration Assay in CM

The Bradford assay kit (Thermo, USA) was employed for rapid protein concentration determination. Protein standards were prepared, and a standard curve was constructed. For each sample, 150 μL of CM was taken and mixed with 250 μL of G250 dye solution for reaction. The absorbance of the samples at the peak wavelength of 595 nm (A595) was measured, and the values were fitted to the standard curve to calculate the protein concentration.

### 2.5. Cell Viability Assay

Cell viability was assessed using the CCK8 following the manufacturer's instructions. DFMSC after CM collection and THP-1-derived macrophage cells (5000 cells/well) were seeded in a 96-well plate. After 24 and 48 h, RPMI-1640 complete culture medium containing 50 μL of CCK8 solution was added. After a 4-h incubation, absorbance at 450 nm was measured.

### 2.6. qRT-PCR

This study used the EZ-press RNA Purification Kit, the Color Reverse Transcription Kit, and the 2× Color SYBR Green qPCR Master Mix to extract total RNA from cells. The RNA was reverse transcribed into complementary deoxyribonucleic acid (cDNA) and analyzed using QuantStudio1 RT-PCR Systems (Thermo Fisher Scientific, USA) for messenger ribonucleic acid (mRNA) levels. Gene expression was quantified using the 2^−*ΔΔ*CT^ method, with GAPDH as internal control.  Table 1 provides qPCR primer sequences.

### 2.7. Immunocytochemistry Assay

Each cell climbing slide was seeded with 5 × 10^5^ macrophages. After 24 and 48 h of experimental grouping, the cells were fixed in precooled methanol (−20°C) for 5 min. Following fixation, cells were permeabilized with 0.3% Triton X-100 for 15 min and blocked with 5% goat serum for 30 min. Primary antibodies, including CD68 (1 : 100), CD80 (1 : 50), and CD206(1 : 50), were incubated with the slides overnight at 4°C. The slides were incubated with fluorescently labeled secondary antibodies the next day, followed by mounting using an antifading mounting medium containing 4′,6-diamidino-2-phenylindole (DAPI). Fluorescence was visualized and analyzed using a Zeiss Axio Vert. A1 microscope and the ZEISS ZEN microscopy software (ZEISS, Germany).

### 2.8. Transcriptome Analysis

#### 2.8.1. RNA Collection and Acquisition of Transcriptome Data

After Trizol-based RNA extraction, we assessed RNA quality using the Qubit RNA HS Assay Kit for quantification and the Agilent 4200 TapeStation system for RNA integrity evaluation (RIN). Libraries were constructed, and their concentration was accurately quantified using Kapa qPCR. The fragment size of the libraries was determined by the Agilent 4200 TapeStation system. After passing quality control, libraries were pooled based on effective concentration and desired sequencing data. Finally, Illumina PE150 sequencing (Illumina, Inc., USA) was carried out.

#### 2.8.2. Transcriptome Data Analysis

We conducted differential gene expression analysis by transforming the Reads Count into Transcripts Per Kilobase of exon model per Million mapped reads (TPM) value using the DESeq2 package (Version 1.12.3) in R Studio (Version 2023.06.0). To annotate and analyze the differentially expressed genes (DEGs), we employed the clusterProfiler package (Version 3.17), which facilitated functional annotation, as well as Gene Ontology (GO) and Kyoto Encyclopedia of Genes and Genomes (KEGG) enrichment analyses. Moreover, we performed an extensive multidatabase analysis, including TRUUST transcription factor enrichment analysis. For this purpose, we utilized the Metascape webpage (https://metascape.org) to conduct a comprehensive synthesis analysis.

### 2.9. Western Blotting

Cellular whole and nuclear proteins were extracted using the Nuclear Extract Kit according to the manufacturer's instructions. A bicinchoninic acid (BCA) protein assay kit quantified and normalized all samples. After heated for 10 min at 95°C, the samples were separated via sodium dodecyl sulfate polyacrylamide gel electrophoresis (SDS–PAGE) and transferred to a polyvinylidene fluoride membrane. The membranes were incubated with antibodies against EGR-1 (1 : 1 000), ERK1/2 (1 : 1000), SAPK/JNK (1 : 1000), P38 (1 : 1000), GAPDH (1 : 1000), and LaminA/C (1 : 1000) at 4°C overnight, followed by horseradish peroxidase (HRP)-conjugated secondary antibodies treated for 1 h. Blots were visualized by Chemiluminescent Kit.

### 2.10. Utilization of the Inhibitor PD98059

The mitogen-activated extracellular signal-regulated kinase (MEK) inhibitor PD98059 (MCE, USA) was employed in experiments designed to block the MAPK-ERK pathway. The powder was solubilized in 1.8707 mL dimethyl sulfoxide (DMSO) to a concentration of 10 mM. Following the induction of THP-1 cells into macrophages, the original medium was removed, the cells were washed three times with PBS, and 10 mM PD98059 was diluted to 10 μM with serum-free RPMI-1640 medium. The THP-1-derived macrophages were induced throughout the process and protected from light for a period of 22 h. Subsequently, the cells were rewashed, and DFMSC-CM induction experiments were conducted.

### 2.11. Statistical Analysis

Data analysis was performed using GraphPad Prism 9 software. A two-tailed unpaired Student's *t*-test was employed to assess differences between groups simultaneously and within the same group at different time points. The significance level was set at *P* value <0.05, indicating a statistically significant difference.

## 3. Results and Discussion

### 3.1. Results

#### 3.1.1. Isolation and Identification of DFMSC

**Figure 1:**
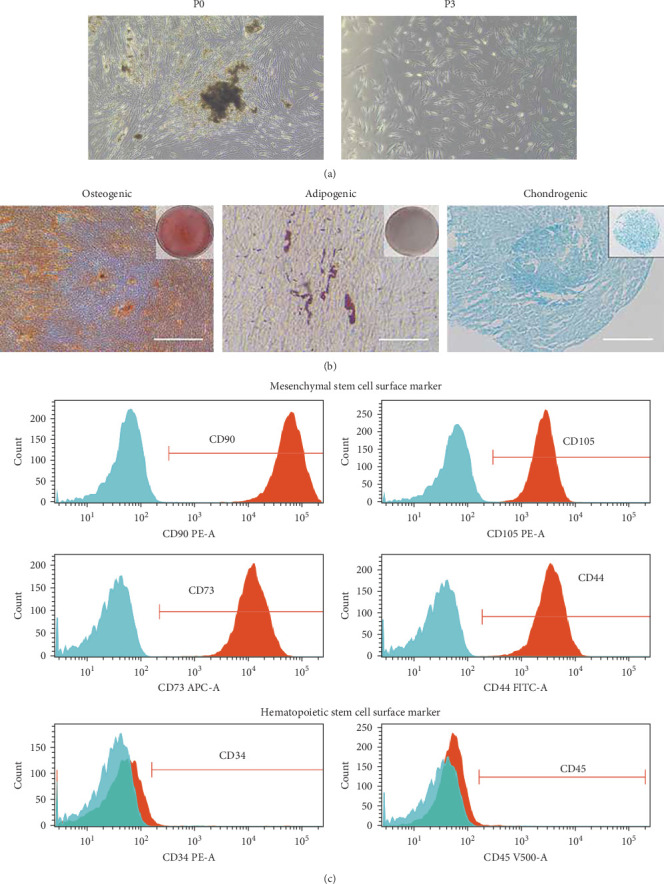
Isolation and identification of DFMSC. (A) Primary cultured DFMSC. The cell morphology of DFMSC in passage 0 and passage 3. (B) The odorogenic differentiation of DFMSC was investigated using Alizarin Red S staining. The adipogenic differentiation of DFMSC was investigated using Oil Red O staining. Chondrogenic differentiation of DFMSC was investigated by Alcian blue staining. (C) Flow cytometry assays showed high levels of the mesenchymal stem cell markers CD90, CD105, and CD73 and low levels of the hematopoietic cell markers CD45 and CD34 in DFMSC. DFMSC, dental follicle-derived mesenchymal stem cell.

Dental follicle-derived cells were extracted from the five volunteers' pericoronal area above the third molars. The participants included three males (aged 16, 19, and 15 years) and two females (aged 18 and 20). The isolated cells demonstrated robust cellular viability and adhered effectively, exhibiting a morphological phenotype reminiscent of MSCs, characterized by elongated, spindle-shaped, and irregular triangular cells (Figure 1A). Subsequently, the successful induction of multilineage differentiation, encompassing osteogenic, adipogenic, and chondrogenic lineages, was achieved. Alizarin Red S and Oil Red O staining successfully demonstrated the presence of mineralized nodules and lipid droplets. Furthermore, Alcian Blue staining facilitated the identification of acid mucopolysaccharides within chondrocytes, as indicated by the appearance of blue staining (Figure 1B). To comprehensively evaluate the cellular characteristics, flow cytometry analysis was conducted in accordance with the guidelines stipulated by the International Society for Stem Cell Research (ISSCR) [27]. This analysis aimed to assess the expression profile of MSC markers. Notably, the isolated cells derived from passages 3–5 exhibited an exceedingly high positivity rate (exceeding 99%) for CD90, CD105, CD73, and CD44, widely acknowledged as reliable markers for MSC identification. Conversely, the positivity rate for CD45 and CD34, markers conventionally associated with hematopoietic cells, was consistently below 1% (Figure 1C). Consequently, the cells isolated from the dental follicle were unambiguously identified as DFMSCs.

#### 3.1.2. DFMSC-CM Preparation Process, Quality Control, and Performance Testing

**Figure 2:**
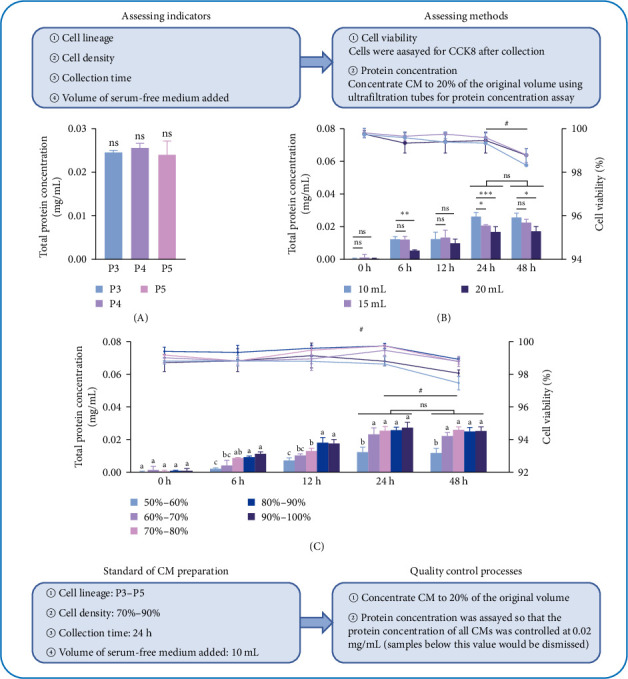
Development of DFMSC-CM preparation protocol based on the effects of different conditions on protein concentration and cell viability. (A) Comparison of protein concentrations in CM extracted from the DFMSC in passages 3–5. (B) Collecting 10, 15, and 20 mL serum-free medium affects DFMSC-CM protein concentration and DFMSC cell viability. (C) The effects of 50%–100% cell density on DFMSC-CM protein concentration and cell viability. The left *Y*-axis corresponds to the bar graph showing total protein concentration, with statistical differences indicated by letter annotations. The right *Y*-axis corresponds to the line graph showing DFMSC cell viability assessed by CCK8, # denotes statistically significant differences between 24 and 48 h, *p*  < 0.05. Data are represented as the mean ± SD (*n* = 3). CCK8, Cell Counting Kit-8; DFMSC-CM, dental follicle-derived mesenchymal stem cell conditioned medium; SD, standard deviation.

To ensure the effectiveness of DFMSC-CM and to minimize differences between experimental groups arising from variations in batches, a systematic exploration of the preparation process was undertaken, accompanied by the implementation of stringent quality control measures. Initially, it was demonstrated that CM derived from the 3–5 passage DFMSCs exhibited consistent protein levels (Figure 2A). Subsequent experimentation focused on the collection of serum-free medium. It was observed that the harvesting of 10 mL resulted in notably higher protein concentrations. Moreover, no significant discrepancy in total protein was observed between collections conducted at 24 and 48-h intervals. Concomitantly, cell viability remained unaltered over the course of 24 h (Figure 2B). Standardized DFMSC-CM was subjected to gradient dilutions in order to assess its impact on macrophage viability and inflammatory cytokine expression under inflammatory conditions. As the concentration of CM decreased, macrophage viability and cytokine expression were affected. Notably, no intrinsic proinflammatory properties were observed during the induction of pure CM (Figure S1). Further investigation involved testing various cell densities, which revealed optimal densities ranging from 70% to 90%. At these densities, the highest protein concentrations were observed without compromising the viability of DFMSCs (Figure 2C). Consequently, a standardized protocol for preparing DFMSC-CM was devised, ensuring a consistent protein concentration of 0.02 mg/mL through meticulous measurements of each batch of concentrated CM.

#### 3.1.3. DFMSC-CM Has an Impact on the Immunoregulatory Function and the Phenotype of THP-1-Derived Macrophages Under Inflammatory Conditions

**Figure 3:**
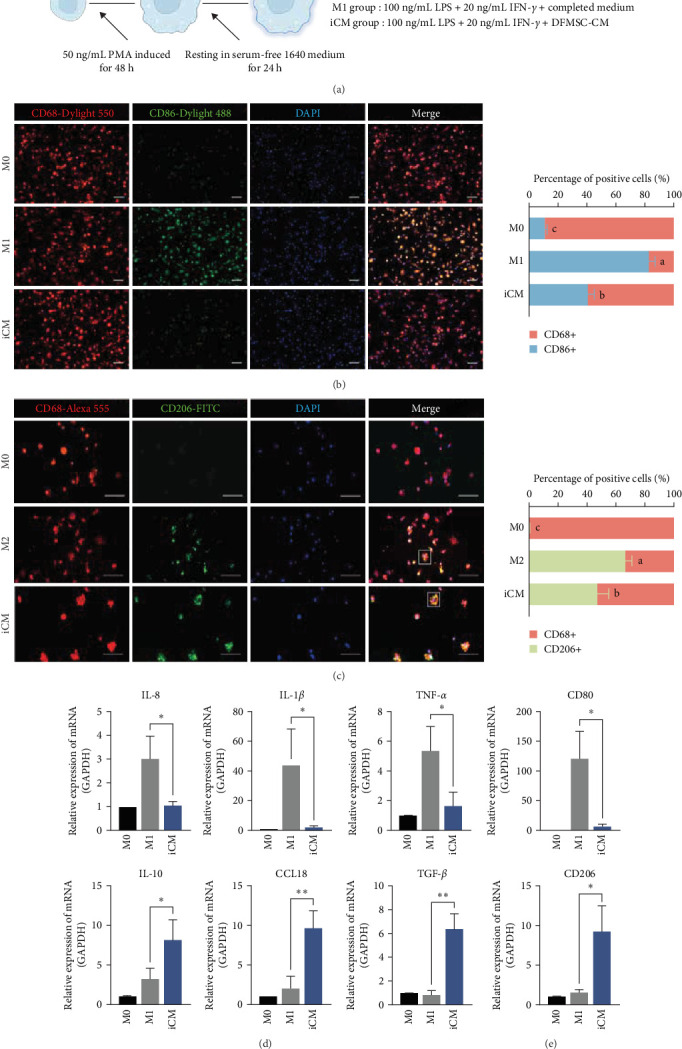
DFMSC-CM facilitates macrophages' conversion to an anti-inflammatory phenotype in vitro under inflammatory conditions. (A) The protocol for inducing macrophages in vitro and the composition of the culture media used for each group. (B, C) Immunofluorescence double staining of CD68 and CD86, along with quantification of the percentage of positive cells. CD68 was labeled with Dylight550 (red), CD86 and CD206 were labeled with Dylight488 (green), and nuclei were stained with DAPI (blue). In merged images, overlapping red and green fluorescence appears yellow. Selected areas outlined in white are presented in the Supporting Information. (D) qRT-PCR analysis of inflammatory cytokines IL-8, IL-1*β*, and TNF-*α*, as well as anti-inflammatory factors IL-10, CCL18, and TGF-*β*. Data are represented as the mean ± SD (*n* = 3), statistical significance is stated *⁣*^*∗*^=*p*  < 0.01, *⁣*^*∗∗*^=*p*  < 0.001 (scale bar = 20 μm). DAPI, 4′,6-diamidino-2-phenylindole; DFMSC-CM, dental follicle-derived mesenchymal stem cell conditioned medium; IL, interleukin; TGF-*β*, transforming growth factor *β*; SD, standard deviation.

When we examined the timing of the addition of DFMSC-CM, we found that the addition of CM at the same time as the induction of M1, that is, the coculture of macrophages with DFMSC-CM in an inflammatory state, had a much better effect on the reduction of inflammatory factors than the addition of CM after the formation of M1 (Figure S2). Thus, we have a well-formed in vitro model of DFMSC-CM-induced macrophages in the inflammatory state (Figure 3A).

The expression of surface markers, including CD80 for M1 macrophages and CD206 for M2 macrophages, was evaluated. The results indicated that the CD80 gene expression in the iCM group was significantly lower than in the M1 group. In comparison to the M1 group, there was a discernible upward trend in CD206 expression within the iCM group (Figure 3E). A double immunofluorescence staining procedure was employed at the protein expression level to assess the expression of CD68 and CD86 and CD68 and CD206. The number of CD68 and CD86 double-positive cells in the iCM group was observed to be significantly lower than that in the M1 group, with the ratio in the iCM group being substantially lower than in the M1 group (Figure 3B). The proportion of CD68+CD206+ double-positive cells was calculated. Although the frequency was lower than observed in the M2 group, it was still detectable in the iCM group (Figure 3C). The oval cells were identified as CD68 and CD206 double-positive M2 macrophages, as confirmed by magnified images and immunofluorescence colocalization pictures in the Supporting Information (Figure S3), it can be observed that the area of red fluorescence of CD68 encompasses the green fluorescence of CD206, indicating that these two markers are expressed by the same cell. This indicates a trend in the iCM group towards forming M2 macrophages. However, it is not possible to completely prevent the formation of M1 cells. A comparison of the gene expression of inflammatory and anti-inflammatory factors between the two groups revealed that, compared to the M1 group, the iCM group exhibited a statistically significant reduction in Interleukin (IL)-1*β* and TNF-*α*, while IL-8 remained relatively stable. Nevertheless, anti-inflammatory factors, such as CCL18 and IL-10, demonstrated statistically significant increases (Figure 3D). These findings indicate that CM enhances macrophages' inflammatory regulatory function within an inflammatory environment and steers their transformation toward inflammation suppression.

#### 3.1.4. Transcriptome Sequencing Reveals an Essential Role for the MAPK Pathway and the Transcription Factor EGR1 in the Regulation of Macrophages

**Figure 4:**
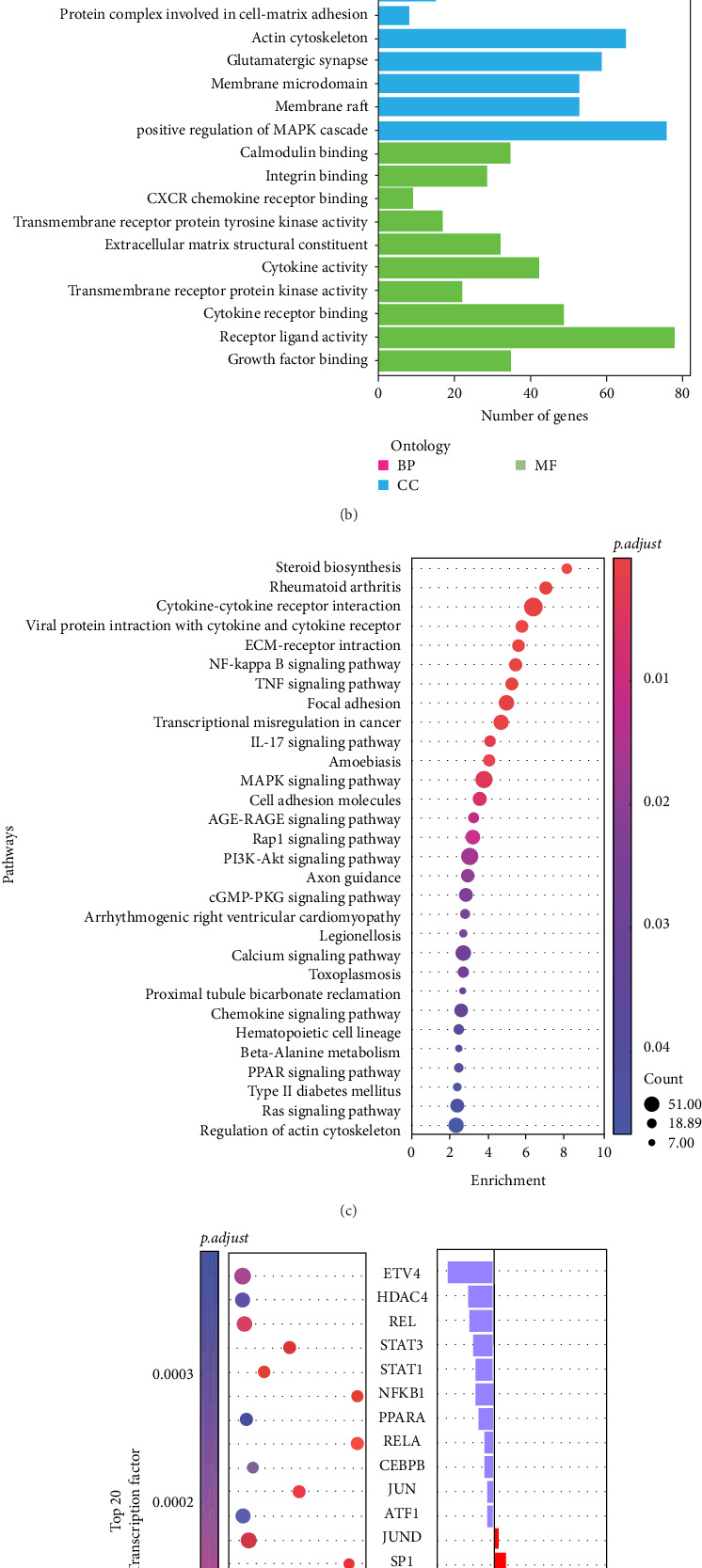
Transcriptome sequencing analysis of M1 and iCM macrophages included. (A) Cluster heatmap and volcano plot for differentially expressed genes. (B) GO enrichment analysis showcasing the top 10 genes in molecular function (MF), biological process (BP), and cell component (CC) sections with *p.adjust* < 0.01. (C) KEGG pathway analysis displaying the top 35 genes in pathways with *p.adjust* < 0.05; dot size indicates enriched gene count, with a redder color signifying higher significance. (D) TRUUST database identified the top 20 transcription factors with *p.adjust* < 0.05 and maximum regulated genes. Transcriptome sequencing Readcount changes for transcription factors are also given as log2FC. (E, F) Western blot determined and quantified the MAPK activation pathway and EGR1 protein expression. Data are represented as the mean ± SD (*n* = 3). Statistical significance is stated *⁣*^*∗∗*^*p*  < 0.001, *⁣*^*∗∗∗∗*^*p*  < 0.00001, ns, no significance. EGR1, early growth response 1; GO, Gene Ontology; KEGG, Kyoto Encyclopedia of Genes and Genomes; MAPK, mitogen-activated protein kinase; SD, standard deviation.

To elucidate the underlying mechanism by which DFMSC-CM regulates macrophage polarization in the presence of inflammation, transcriptomic sequencing was conducted on macrophages from two distinct groups, M1 and iCM. Using rigorous criteria of |log2 (FoldChange)| > 1 and *p.adjust* < 0.05, we identified a total of 3649 genes that were differentially expressed, with 2789 genes upregulated and 860 genes downregulated in the iCM group compared to the M1 group (Figure 4A). The clusterProfiler package was employed for gene annotation and GO, KEGG enrichment analysis [27].

The GO and KEGG databases were found to enrich DEGs in the MAPK signaling pathway (Figure 4B,C). Moreover, the Metascape multidatabase analysis revealed that the MAPK cascade was also enriched. Western blotting was employed to identify the three principal activation pathways of MAPK, namely, ERK1/2, p38, and SAPK/JNK. The expression of ERK1/2 was the most pronounced, with a notable difference in expression between the M1 and iCM groups (Figure 4E). A transcription factor enrichment analysis was conducted using the TRUUST database to identify the transcription factors regulating the DEGs. The top 20 transcription factors are listed in the figure, including classical inflammation-related transcription factors such as NFKB1, JUN, REL, and others. However, the expression of these transcription factors exhibited a decreasing trend. While EGR1, which exhibited a considerable increase in expression (Figure 4D), attracted our attention, there were fewer DEGs regulated by EGR1. Nevertheless, among the DEGs were those related to inflammation initiation, including IL-6, CXCL8, and TNF (Figure S4). Furthermore, EGR1 protein expression was significantly higher in the iCM group than in the M1 group (Figure 4F).

#### 3.1.5. Regulation of Macrophage Polarization by DFMSC-CM Acts Through the ERK1/2-EGR1 Axis

**Figure 5:**
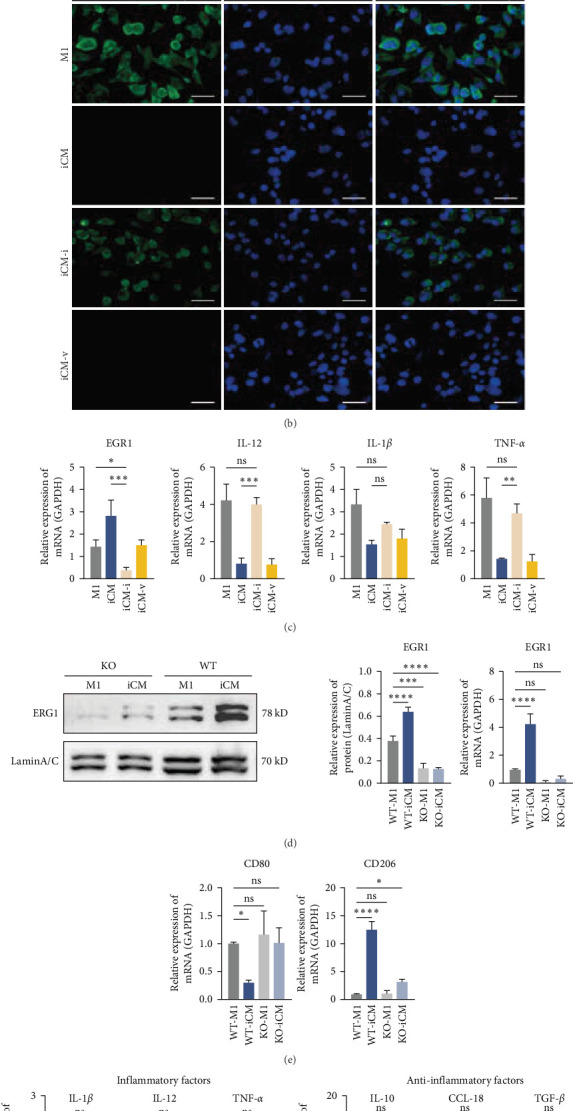
DFMSC-CM regulates macrophages through the MAPK-ERK1/2-EGR1 axis. (A) Western blot results of whole-cell ERK1/2, nuclear phosphorylated ERK1/2 (p-ERK1/2), and EGR1 in M1, iCM, iCM-i (iCM with PD98059 inhibitor), and iCM-v (iCM with DMSO vehicle) groups. GAPDH, LaminA/C, were used as internal references ERK1/2, p-ERK1/2, and EGR1. (B) Immunofluorescence staining results in the M1, iCM, iCM-i, and iCM-v groups, CD86 was labeled green by Dylight488, and the nuclei were labeled blue by DAPI. (C) Gene expression levels of the transcription factor EGR1 and the inflammatory factors IL-10, IL-1*β*, and TNF-*α*. The internal reference gene was GAPDH. (D) EGR1 knockdown THP-1-derived macrophage knockdown efficiency assay through Western blot and qRT-PCR. (E,F) RT-qPCR detection of polarization-related gene expression. Data are represented as the mean ± SD (*n* = 3). Statistical significance is stated *⁣*^*∗*^*p*  < 0.01, *⁣*^*∗∗*^*p*  < 0.001, *⁣*^*∗∗∗*^*p*  < 0.0001. *⁣*^*∗∗∗∗*^*p*  < 0.00001 (scale bar = 20 μm). DAPI, 4′,6-diamidino-2-phenylindole; DFMSC-CM, dental follicle-derived mesenchymal stem cell conditioned medium; DMSO, dimethyl sulfoxide; EGR1, early growth response 1; ERK, extracellular regulated protein kinases; GAPDH, glyceraldehyde-3-phosphate dehydrogenase; IL, interleukin; MAPK, mitogen-activated protein kinase; SD, standard deviation.

A synergistic change was observed in the temporal expression of ERK1/2 and EGR1 at 6, 12, and 24 h, prompting an investigation into a possible regulatory relationship between the two (Figure S5). To investigate this further, the MAPK-ERK1/2 pathway inhibitor PD98059 was added. In the experimental group in which the pathway was blocked (iCM-i), there was a significant reduction in ERK1/2 at the whole-cell level compared to the iCM group. The phosphorylated ERK1/2 level and nuclear EGR1 also demonstrated a downward trend, comparable to the expression levels observed in the M1 group (Figure 5A). The changes in gene expression levels were consistent with the protein expression patterns, with an increase in relative gene expression. When the MAPK-ERK1/2-EGR1 axis was disrupted, the proinflammatory M1 macrophage surface marker CD86 was detected. A comparison of the iCM group with the empty vector group (iCM-v) revealed the presence of a CD86 signal (labeled with green fluorescence) in the iCM-i group. Moreover, the expression of proinflammatory factors, including IL-12, IL-1*β*, and TNF-*α*, was upregulated.

To further substantiate the role of the transcription factor EGR1, an EGR1 knockout THP-1 cell line was employed (Figure 5D). The deletion of EGR1 resulted in the upregulation of inflammatory factors previously suppressed by DFMSC-CM. The expression of IL-1*β*, IL-2, and TNF-*α* in the KO-iCM group was consistent with that observed in the wild-type M1 group, while the KO-M1 group did not show significant differences from the wild-type group. Notably, the low expression levels of some anti-inflammatory factors, such as IL-10, CCL-18, and TGF-*β*, were not statistically different from those observed in the wild-type M1 group (Figure 5F). The altered secretory function of macrophages indicated a change in the polarization type of the macrophages. Consequently, the polarization markers CD80 and CD206 were examined. The results demonstrated that the CD80 gene expression in the KO-iCM group was elevated, whereas the expression of CD206, which was initially high, was diminished (Figure 5E). These findings suggest that EGR1 is a crucial factor in the DFMSC-CM's inhibition of inflammation and plays a bidirectional role in initiating the anti-inflammatory function of macrophages.

### 3.2. Discussion

Our research findings align with previous investigations into the indirect coculture of DFMSC and immune cells, underscoring the immunomodulatory potential of DFMSC. DFMSCs, originating from dental follicles, exhibit tissue regenerative potential and immunomodulatory functions [28]. As oral MSCs, they offer advantages in availability and minimal ethical concerns compared to other dental sources. In contrast to the limited tissue mass of deciduous tooth stem cells (stem cells from human exfoliated deciduous teeth [SHED]), the complex extraction of dental pulp stem cells (DPSCs), and the problematic isolation of periodontal ligament stem cells (PDLSCs), DFMSC, found in the soft tissue around the underdeveloped tooth, can be directly cultured after isolation. Previous research has shown that tooth eruption, characterized by the emergence of teeth, is closely linked to the bone-resorbing function of osteoclasts in the adjacent dental tissues, with DFMSC playing a vital role in this process. Complementing this process by releasing colony-stimulating factor-1 (CSF-1) and monocyte chemoattractant protein-1 (MCP-1), DFMSC attracts a substantial influx of monocytes into the dental follicle tissue [29, 30]. Monocytes differentiate into osteoclasts, effectively creating a pathway over the tooth germ and facilitating the smooth eruption of the tooth by clearing the way for it to emerge. Recently, researchers have harnessed the potential of DFMSCs in studies related to immune regulation, as MSCs exhibit paracrine regulation in inflammation [31–34]. Their CM impact LPS-induced inflammatory dental pulp, enhancing anti-inflammatory factors and proteins related to osteogenesis, angiogenesis, neurogenesis, and antiapoptosis [24]. The study by Chen et al. [23] also indicated that DFSC-CM facilitates macrophage M2 polarization by secreting TGF-*β*3 and TSP-1. From the perspective of the experimental findings, our study aligns with the conclusions of the aforementioned study. However, our study delves deeper into the molecular pathway alterations in macrophages following CM treatment, whereas the aforementioned study primarily elucidates the specific components of CM that influence macrophages. It is noteworthy that the study reported that TGF-*β*3 was able to activate the TGF*β* signaling pathway. Furthermore, there was a highly correlated crosstalk between the MAPK pathway and the TGF*β* pathway through the activation of the membrane receptor, which provided additional evidence for the activation of the MAPK-ERK pathway in the experimental results [35].

Our study also reveals alterations in macrophage intracellular pathways in response to DFMSC-CM regulation. The MAPK-ERK1/2 pathway and its associated transcription factor, EGR1, are particularly interesting. Located in the q31.1 cytokine gene cluster region of human chromosome 5, the EGR1 gene is usually rapidly induced by growth factors, cytokines, and other stimuli, exhibiting its “early response” characteristics. EGR1 was considered a preinflammation marker for a considerable period, detected in macrophage-related inflammation processes [36, 37]. However, as researchers delved into the formation and polarization of macrophages, it became evident that EGR1 plays a pivotal role in the transition of monocytes to macrophages within the mononuclear phagocyte system (MPS) [38, 39]. It was not until a groundbreaking study by Macro et al. that a decisive understanding was achieved [40]. They found that while EGR1 does regulate gene expression during the transition from monocytes to macrophages, it subsequently exerts inhibitory effects that suppress the transcription of inflammatory genes. The identification of novel EGR1 binding sites at a large set of inflammatory enhancers, even in the absence of its binding motif, is presented. It is demonstrated that EGR1 repressive activity suppresses inflammatory genes and is mediated by the NuRD corepressor complex. It has also been shown that DNA damage-inducible transcript 3 (DDIT3) promotes M1 polarization by inhibiting EGR1; in other words, EGR1 uninhibited prevents M1 polarization [41]. In mouse bone marrow-derived macrophages, small interfering ribonucleic acid (siRNA)-mediated EGR1 knockdown significantly attenuated IL4-induced expression of Itgax, Nipal1, Bhlhe40, CD206, and Ffar4, underscoring EGR1′s pivotal role in the polarization of M2-type macrophages [42]. EGR1, which are also considered genetic hubs of divergent bacillary infections for 24 h, enable macrophages to survive the harsh conditions of bacterial infections, which further explains the finding of our study that DFMSC-CM can maintain the cellular activity of macrophages in inflammatory states [43].

**Figure 6:**
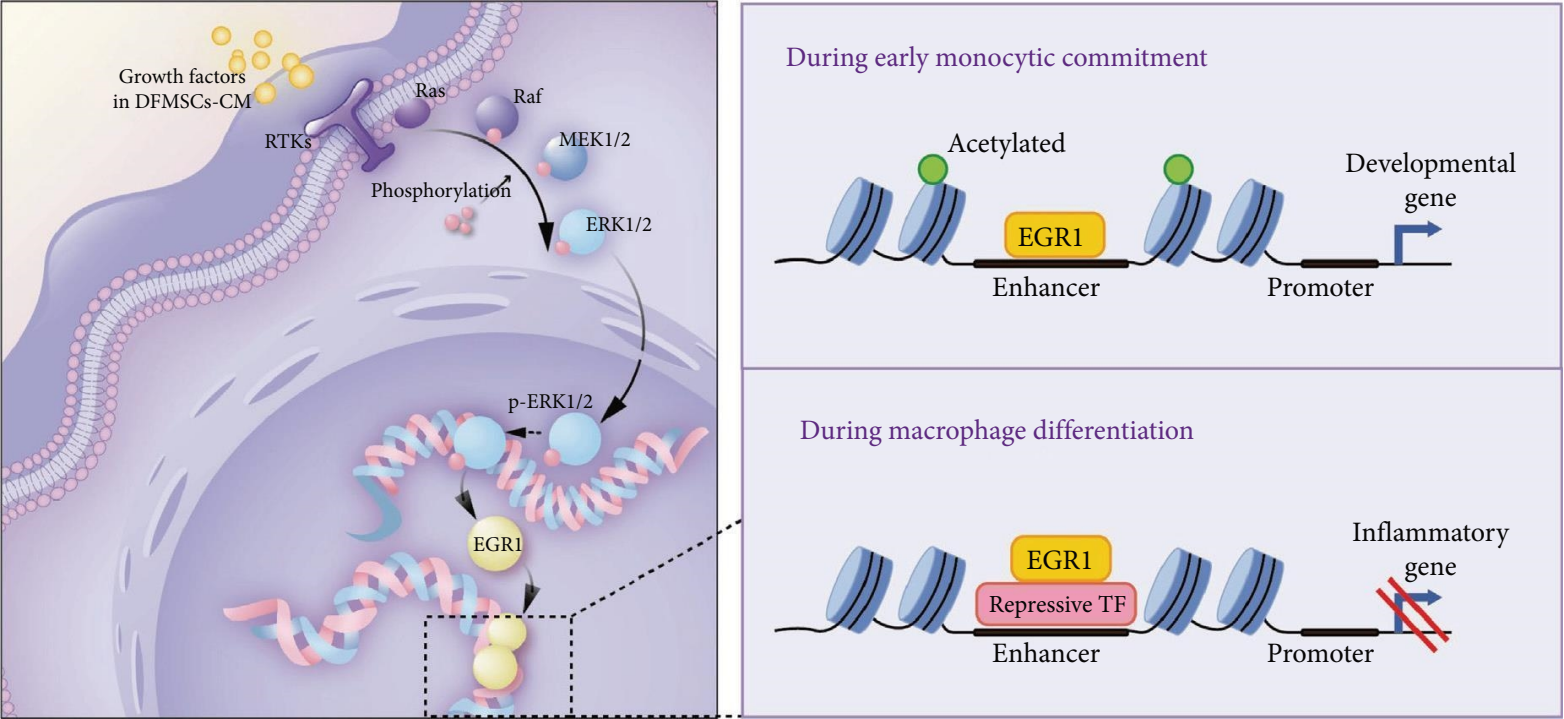
The schematic diagram of the molecular mechanism of DFMSC-CM regulation on macrophages. The components of DFMSC-CM activate receptor tyrosine kinases (RTKs) on THP-1-derived macrophages, initiating the MAPK signaling pathway through the ERK pathway. Subsequently, RAS-Raf-MEK1/2-ERK1/2 undergoes phosphorylation, and phosphorylated ERK1/2 (p-ERK1/2) translocates into the nucleus, upregulating the transcription factor EGR1. In regulating inflammation, EGR1 plays a role in inhibiting the expression of inflammation-related genes in macrophages, thereby modulating the phenotype and function of macrophages. During the differentiation of monocytes into macrophages, EGR1 typically upregulates the expression of development-related genes. DFMSC-CM, dental follicle-derived mesenchymal stem cell conditioned medium; EGR1, early growth response 1; ERK, extracellular regulated protein kinases; MAPK; mitogen-activated protein kinase; MEK, mitogen-activated extracellular signal-regulated kinase.

Furthermore, a robust association has been demonstrated to exist between the MAPK signaling pathway and EGR1. Researchers have consistently reported an association between EGR1 and the MAPK cascade pathway [44, 45]. Activation of endoplasmic reticulum stress triggers the MAPK pathway, and the Sarcoma gene, a proto-oncogene (SRC)-Rat sarcoma viral oncogene (RAS)-Rapidly Accelerated Fibrosarcoma (RAF)-MEK-ERK cascade mechanism leads to enhanced phosphorylation of the transcription factor ELK1, which constitutively binds to the EGR1 promoter along with serum response factor (SRF) and is phosphorylated by the nuclear-localized ERK, thereby enhancing EGR1 expression [46, 47]. IL-4 stimulation increases the ERK pathways, with EGR1 further regulating downstream gene expression [48]. IL-13 promotes the binding of EGR1 to the EGR1 binding sequence (EBS) in the promoter region of kallikrein-related peptidase 7 (KLK7) through activation of the ERK1/2 MAPK pathway, thereby initiating transcription and expression of the KLK7 gene [49]. Drugs have also been reported to block macrophage polarization to M1 cells via an alpha7nAChR/ERK/Egr-1 pathway feedback pathway [50]. The secretome of MSC has been found to contain a plethora of cytokines, and the activation of the MAPK pathway by DFMSC-CM has been well-documented, as it plays a crucial role in various cellular physiological activities such as cell growth, development, and differentiation. Among the three activation pathways of MAPK, the ERK1/2 pathway is associated explicitly with growth factors [51, 52]. It is noteworthy that MSCs have the ability to secrete growth factors. An overview of cytokines reveals that MSC-secreted growth factors, classified as proinflammatory cytokines, paradoxically induce anti-inflammatory polarization in macrophages, likely mediated through the ERK1/2 pathway [53, 54]. Previous studies have reported the activation of ERK1/2 in macrophages through paracrine signaling by bone marrow MSCs, adipose stem cells, and other types of MSC [55–57]. Synthesizing existing research knowledge with our findings, we have delineated a mechanism in Figure 6: DFMSC-CM activates RTKs on macrophages, initiating the MAPK pathway via ERK. Sequentially, RAS-RAF-MEK-ERK phosphorylates and translocates p-ERK1/2 into the nucleus, upregulating EGR1. During monocyte-to-macrophage transformation, EGR1 shifts to upregulate genes associated with development during the orchestration of inflammation regulation.

However, our results identified a shortcoming of the utilization of DFMSC-CM. Despite its superior anti-inflammatory effect and induction of M1-type cells, it failed to enhance macrophage polarization or inflammatory state once M1-type cells formed. This is related to the rule of cell polarization in the resting state that, regardless of the type of polarization they are oriented, they must start with M0-type cells and cannot directly interconvert between M1 and M2 types [58, 59]. In recent years, the concept of repolarization has emerged, suggesting that M1- and M2-type cells can transition between each other under specific conditions. Bossche et al. found that mouse and human M1 macrophages were challenging to convert to M2 cells in vitro and in vivo due to mitochondrial dysfunction, whereas M2 macrophages were more plastic and readily repolarized to an inflammatory M1 state [60, 61]. In the pathological setting of inflammation, M2 cells in macrophage repolarization show low tolerance to LPS reprocessing. This explains why most inflammation is directed toward tissue destruction when a bacterial infection is involved [62]. Breaking the imbalance of macrophage repolarization has become one of the new focuses of researchers in controlling chronic inflammation. The investigators inhibited the repolarization of M2 cells to M1 cells by modulating macrophage metabolism, reprogramming transcription factors (e.g., EGR1, as highlighted in our study) to regulate epigenetics, and influencing the immunoregulatory activities of macrophages, thereby promoting disease regression [63–66].

It should be noted at the outset that our study has been subject to limitations. Firstly, despite the previous research by Dong et al., which briefly explored its components, the significant components of DFMSC-CM associated with immune regulation are still a puzzle [67]. Additionally, we have not delved into the specific binding sites of EGR1 regulation and how it regulates the reduction in expression of downstream inflammatory factors. Given that MSCs are well-documented to affect macrophage polarization, future research will likely explore their influence on macrophage repolarization. This could stabilize and permanently regulate macrophages and their inflammatory microenvironment, establishing MSCs and their derivatives as stable immunosuppressive agents. Aiming to contribute to a better understanding of the role of oral-derived MSC in immune regulation, we anticipate that our findings will offer new insights and approaches for developing therapeutic strategies in related fields.

## 4. Conclusions

In our study, we successfully isolated DFMSC from human dental follicles. By coculturing macrophages with DFMSC-CM, we observed a reduction in the pro-inflammatory M1 phenotype and an increase in the anti-inflammatory M2 phenotype of macrophages in inflammatory conditions. Moreover, the immunoregulatory function of macrophages shifted towards inflammation suppression. Through transcriptome sequencing, we discovered that CM enhanced the expression of the transcription factor EGR1 by modulating the MAPK-ERK1/2 signaling pathway, thereby controlling the phenotype and function of macrophages.

## Figures and Tables

**Table 1:** Primer sequences were used in the experiment.

Gene	Primer type	Primer sequences
CD80	Forward primer	TGCCTGACCTACTGCTTTGC
	Reverse primer	AGGGCGTACACTTTCCCTTC

CD206	Forward primer	GATTGCAGGGGGCTTATGGG
	Reverse primer	CGGACATTTGGGTTCGGGAG

IL-10	Forward primer	CTGAGAACCAAGACCCAGACA
	Reverse primer	AAAGGCATTCTTCACCTGCTCC

IL-1*β*	Forward primer	CCAAACCTCTTCGAGGCACA
	Reverse primer	AGCCATCATTTCACTGGCGA

CCL18	Forward primer	AGCTCTGCTGCCTCGTCTAT
	Reverse primer	CGGCCTCTCTTGGTTAGGAG

IL-8	Forward primer	CATGAACGGCAAACTTGGGG
	Reverse primer	AGCAAGCCACTTCTCCTGTC

TNF-*α*	Forward primer	CTGGGCAGGTCTACTTTGGG
	Reverse primer	CTGGAGGCCCCAGTTTGAAT

TGF-*β*	Forward primer	GCAACAATTCCTGGCGATACC
	Reverse primer	ATTTCCCCTCCACGGCTCAA

RELA	Forward primer	CTTCCAAGAAGAGCAGCGT
	Reverse primer	ACGTTTCTCCTCAATCCGGT

NFKB1	Forward primer	ACCGCCACCCGGCTTC
	Reverse primer	GGATGCATTGGGGGCTTTAC

JUN	Forward primer	GTCCGAGAGCGGACCTTATG
	Reverse primer	CTTTTTCGGCACTTGGAGGC

EGR1	Forward primer	TGACCGCAGAGTCTTTTCCT
	Reverse primer	TGGGTTGGTCATGCTCACTA

STAT3	Forward primer	CATCCTGAAGCTGACCCAGG
	Reverse primer	TCCTCACATGGGGGAGGTAG

GAPDH	Forward primer	AATGGGCAGCCGTTAGGAAA
	Reverse primer	GCGCCCAATACGACCAAATC

Abbreviations: EGR1, early growth response 1; GAPDH, glyceraldehyde-3-phosphate dehydrogenase; IL, interleukin; TGF-*β*, transforming growth factor *β*.

## Data Availability

The raw data of transcriptome sequencing used to support the findings of this study have been deposited in the NCBI database repository PRJNA1050846.

## Supporting Information

Supporting Information Figure S1: The gradient dilution of the obtained CM and cocultured gradient diluted CM with macrophages in the inflammatory state were performed. The usefulness of the CM was evaluated by CCK8 assay and gene expression assay of inflammatory factors. Figure S2: Explanation of why we chose to add CM along with LPS and IFN-*γ*. On the one hand, it demonstrates the limited ability of CM to modulate macrophages after LPS and IFN-*γ* induction. On the other hand, the low expression of inflammatory factors in the CM group demonstrates that CM itself does not have the property to cause inflammation. Figure S3: M0 cells induced by IL4 and IL13 together for 48 h can form M2 cells, which is used as a positive control to demonstrate that THP-1-derived macrophages polarized to M2 cells exhibit CD68+CD206+ double-positive omelette-like cells. Figure S4: Complementary to the results of the bioinformatics analysis, the top 100 gene functions enriched by the Metascape database, the results of the protein interaction analysis of the top 10 gene functions, and the Sankey bubble diagram of the top 5 transcription factors with their regulatory genes. Figure S5: Using Western blot analysis, we examined the protein expression of ERK1/2 and EGR1 at the whole-cell level in M0, M1, and iCM groups at 6, 12, and 24-h time points. This allowed us to explore the dynamic changes in these vital signaling molecules during macrophage polarization and upon treatment with DFMSC-CM.
