# Supplementary material for: Conditioned Medium Derived From Human Dental Follicle Mesenchymal Stem Cells Alleviates Macrophage Proinflammatory Responses Through MAPK-ERK-EGR1 Axis
Source: Stem Cells Int. 2024 Nov 29;2024:5514771. doi: 10.1155/sci/5514771 (PMC11623994; doi:10.1155/sci/5514771)
Supplement: Supporting Information — Figure S1: The gradient dilution of the obtained CM and cocultured gradient diluted CM with macrophages in the inflammatory state were performed. The usefulness of the CM was evaluated by CCK8 assay and gene expression assay of inflammatory factors. Figure S2: Explanation of why we chose to add CM along with LPS and IFN-γ. On the one hand, it demonstrates the limited ability of CM to modulate macrophages after LPS and IFN-γ induction. On the other hand, the low expression of inflammatory factors in the CM group demonstrates that CM itself does not have the property to cause inflammation. Figure S3: M0 cells induced by IL4 and IL13 together for 48 h can form M2 cells, which is used as a positive control to demonstrate that THP-1-derived macrophages polarized to M2 cells exhibit CD68+CD206+ double-positive omelette-like cells. Figure S4: Complementary to the results of the bioinformatics analysis, the top 100 gene functions enriched by the Metascape database, the results of the protein interaction analysis of the top 10 gene functions, and the Sankey bubble diagram of the top 5 transcription factors with their regulatory genes. Figure S5: Using Western blot analysis, we examined the protein expression of ERK1/2 and EGR1 at the whole-cell level in M0, M1, and iCM groups at 6, 12, and 24-h time points. This allowed us to explore the dynamic changes in these vital signaling molecules during macrophage polarization and upon treatment with DFMSC-CM. [file 5514771.f1.pdf]

Supplementary Materials

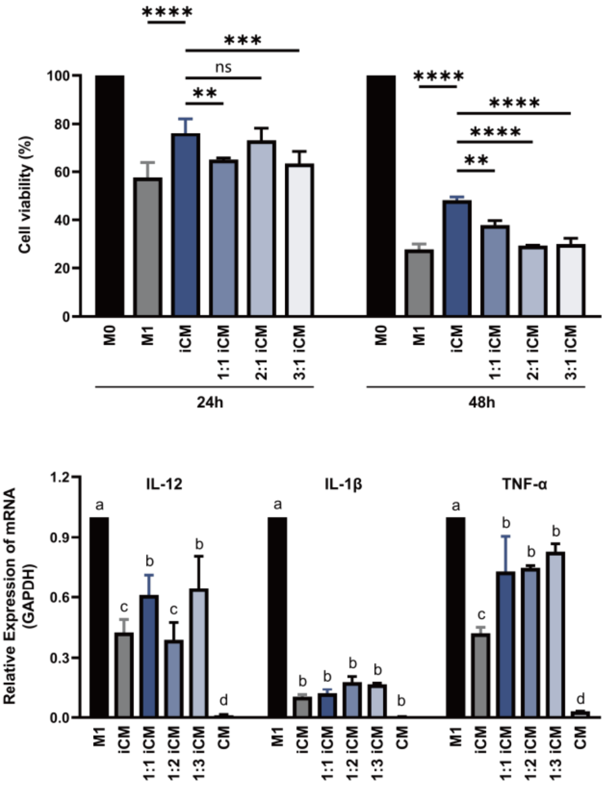

Figure S1: DFMSC-CM Gradient Dilution Modulates Cell Activity Assay and Inflammatory Factor Gene Expression Assay in Macrophages in the Inflammatory State. CM was diluted with RPMI-1640 medium. The initial concentration of CM had a maintenance effect on macrophage activity, which was already significantly higher in the iCM group than in the M1 group and some of the CM dilution groups at 24 hours and substantially different from the other gradient dilution groups at 48 hours. As for the inhibition of inflammatory gene expression, the iCM group significantly inhibited the expression of IL-1, IL-1β, and TNF-α, especially for TNF-α.

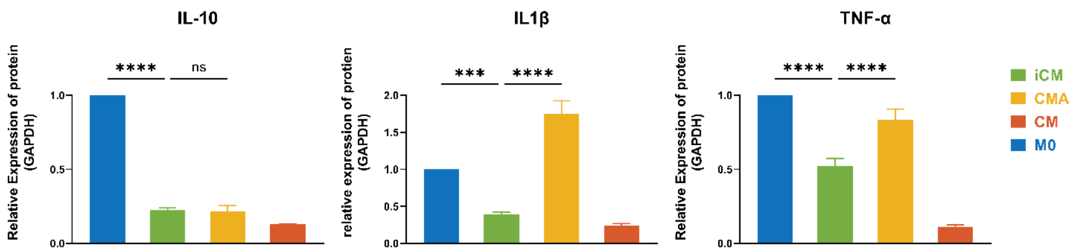

Figure S2: The relative expression levels of inflammatory factor genes IL-10, IL-1β, and TNF-*α*. Inflammatory factor genes were examined, with GAPDH as the internal reference gene. The iCM group received simultaneous treatment with LPS, IFN-γ, and the conditioned medium (CM). After inducing M0 cells into M1 with LPS and IFN-γ for 24 hours, the CMA group was washed with PBS three times before adding CM. The CM group received CM without LPS and IFN-γ. The inflammatory factor genes in the CMA group did not undergo downregulation, suggesting suboptimal therapeutic effects when CM was added after the induction of M0 cells into M1. Simultaneously, this implies that after THP-1-derived macrophages are induced into M1, mesenchymal stem cells face challenges in reversing their phenotype through paracrine means.

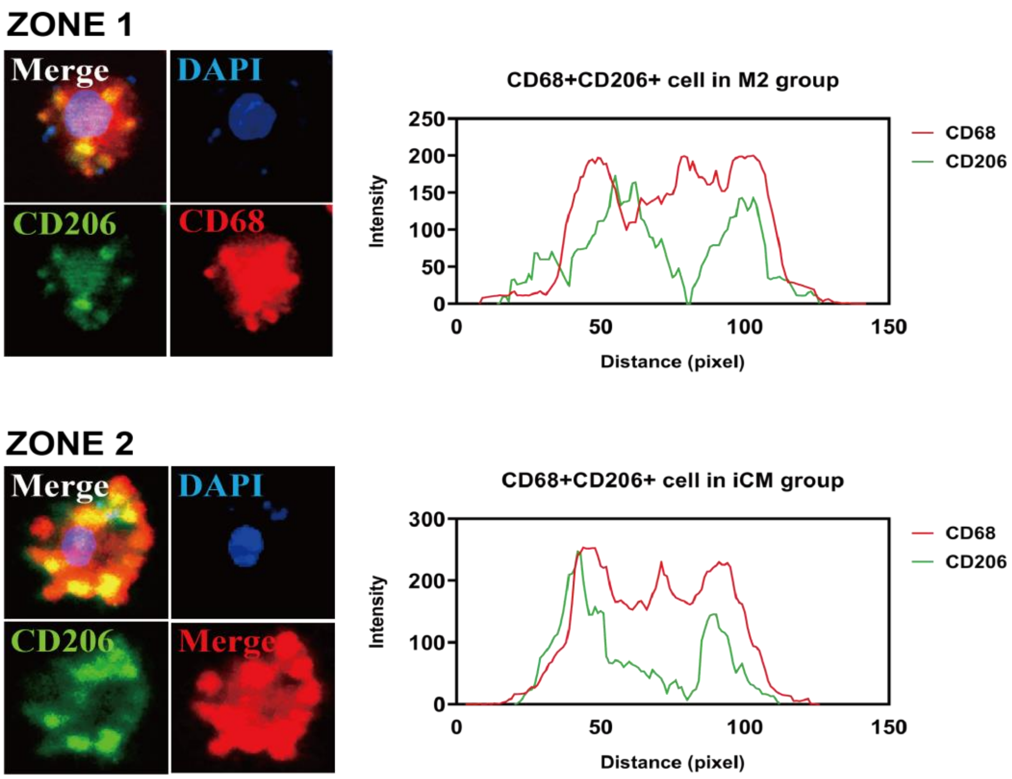

Figure S3: Enlarged images of framed regions in immunofluorescence double staining and fluorescence co-localization analysis of iCM and M2 groups. The cells in both zones are more rounded, with CD68 distributed in the cytoplasm, while CD206 shows a punctate distribution. The co-localization results showed that the CD206 signal was within the range of the CD68 signal, suggesting that they are expressed by the same cell.

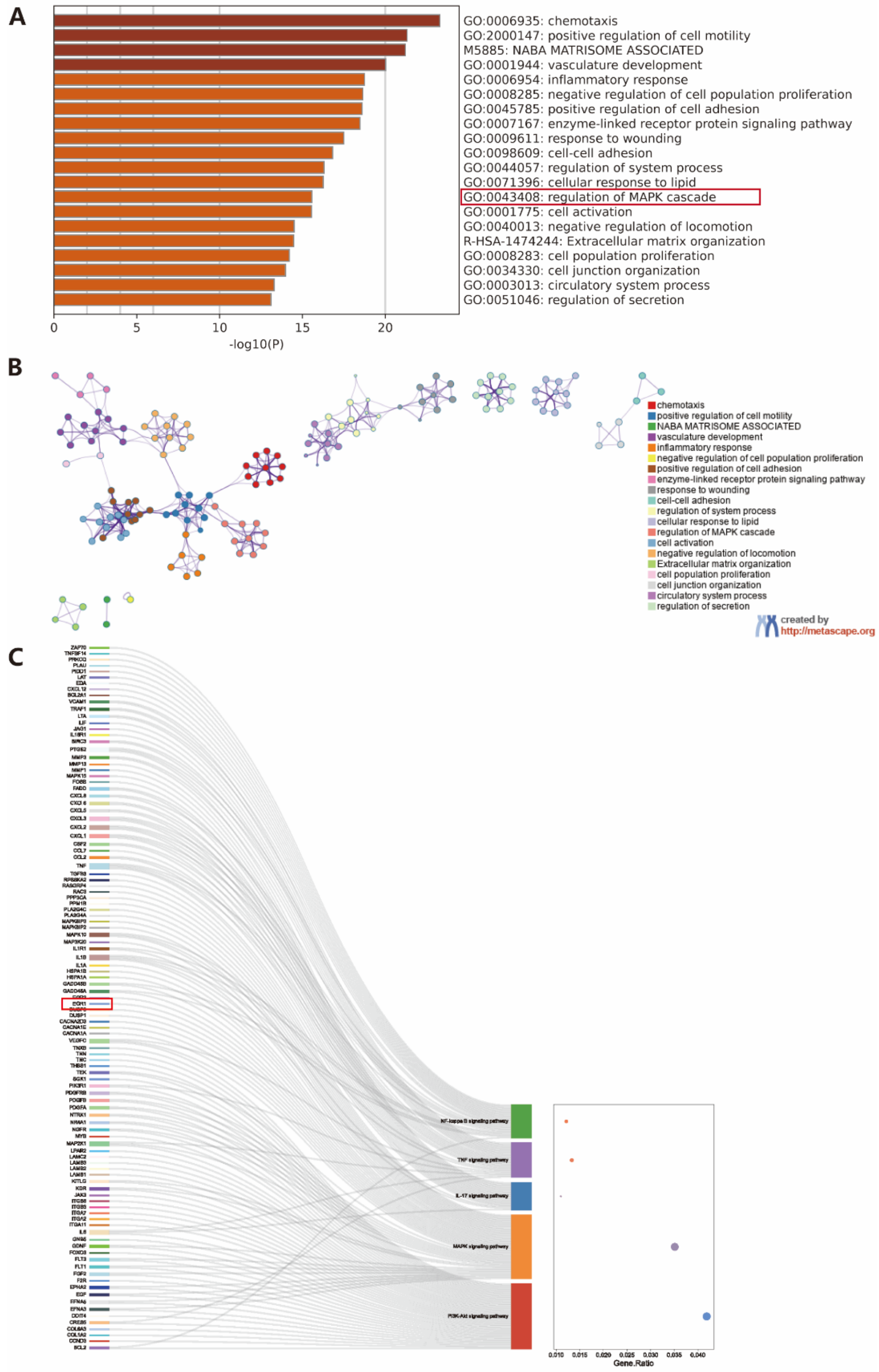

Figure S4: Transcriptome data combined with the results of the comprehensive analysis of multiple Metascape databases. (A) GO analysis filtered the enrichment results with p.adjust < 0.01 and top 20 genes, and regulation of MAPK cascade is listed as ranking 13. **(B)** Protein relationship network visualization results of GO analysis. **(C)** Sankey bubble plot shows the transcription of related genes in each pathway, filtering some genes with p.adjust < 0.01, it can be seen that the MAPK signaling pathway regulates the expression of more genes, and more notably, EGR1(boxed in red) is among them.

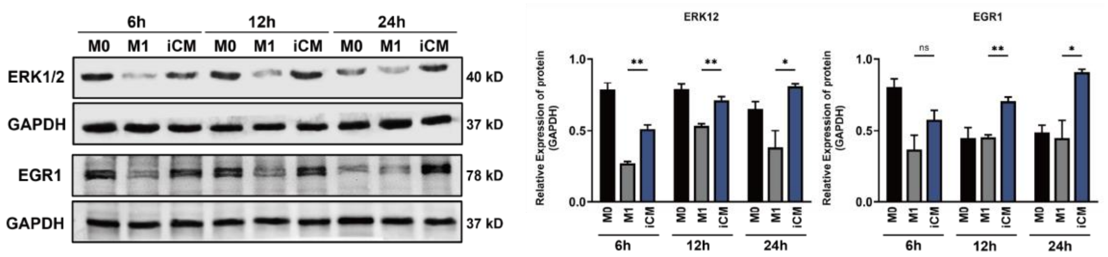

Figure S5: ERK1/2 and EGR1 protein expression in M0, M1, and iCM groups at the whole cell level at hours 6, 12, and 24 using Western blot. Differences in ERK1/2 and EGR1 protein expression appeared at 12 hours and maintained a trend toward sustained high levels at 24 hours. Quantitative analysis of the bands showed that this trend was statistically significant.
